# Secondary Mania in Older Adults: a case report and review of literature

**DOI:** 10.1192/j.eurpsy.2023.1479

**Published:** 2023-07-19

**Authors:** N. El Moussaoui

**Affiliations:** 1 Psychiatry, Arrazi Hospital of Sale, Salé, Morocco

## Abstract

**Introduction:**

Although mania is commonly associated with bipolar disorder, it can have many etiologies. Thus, “primary mania” results from bipolar disorder, whereas “secondary mania” results from pharmacological, metabolic, or neurologic causes. Older adults are at risk for secondary mania because of increased medical comorbidities and neurological changes. In one retrospective study of 50 patients with mania who were older than 65 years, it was the first manic episode for 28% of the patients and 71% had a comorbid neurological disorder.

**Objectives:**

The etiology of mania is important because although acute symptomatic treatment of both primary and secondary mania may be similar, appropriate treatment of secondary mania includes addressing the cause. We present here two case histories of secondary mania in older adults, discuss their presentations and differential diagnosis in turn, and discuss treatment.

**Methods:**

We will present a clinical case of a patient. Ms. A, a 63-year-old divorced woman with no prior medical or psychiatric history, was seen for an acute manic episode with mixed features. She was in her usual state of health until 2 weeks before admission, when she presented with a status epilepticus requiring a one-week hospitalization in the neurology département and treated by Carbamazepine. She then developed an abnormally excited and labile mood, motor excitability accompanied by rapid thoughts, with a total loss of desire and pleasure and thoughts of death.

**Results:**

In our study, the patient presented a manic episode with mixed characteristics secondary to a status epilepticus.

The patient was treated both somatically and psychologically, and the evolution was positive. The patient was stabilised after being put on an antiepileptic drug associated with an antipsychotic drug.

In our study, the patient presented with a manic attack with mixed characteristics secondary to a status epilepticus.The patient was treated both somatically and psychologically, and the evolution was favourable. The patient was stabilised after being put on an antiepileptic drug associated with an antipsychotic drug.

**Conclusions:**

Late onset psychiatric disorders often represent a challenge for the psychiatrists as far as the diagnosis and management issues are concerned. The elderly patients have generally been reported to have associated medical, especially neurological illnesses and are also prone to side effects of various psychotropic medications.

**Disclosure of Interest:**

None Declared

